# Use of educational intervention in reduction of the rate of bloodstream infection associated with the central venous catheter in intensive care unit of adults: integrative review

**DOI:** 10.1186/1753-6561-5-S6-P56

**Published:** 2011-06-29

**Authors:** RA Lacerda, JM Jardim, BN Kosar

**Affiliations:** 1 Surgery-Medical, Escola de Enfermagem da USP - EEUSP, São Paulo, Brazil

## Introduction / objectives

Integrative review with the aim to identify which the educational programs are more effective in reduction of rate in bloodstream infection associated to central venous catheter in intensive care unit of adults.

## Methods

The study was realized using DeCs (Descriptors in Health Science) and MeSH (Medical Subject Headings): Catheter-Related Infections, Hospital Infection’s, Intensive Care Units and Education Continuing. The theorical-practical reason was developed in researches on database *Scielo-Scientific*, *Eletronic Library Online*, Embase, *Cochrane*, *PubMed*, LILACS – Latin American literature and Carribean in Health Science, that was realized a data collection between 2001 and 2010, in indexed journals.

## Results

The search identified 156 abstracts, but with the inclusion and exclusion criteria, only 13 articles were selected. The hand hygiene was part of the educational program in the most studies, as the adequate use the maximum barrier of protection, preference for access to subclavian vein, dressing impregnated with chlorhexidine, impregnated catheter, unnecessary catheter removal and skin antisepsis with chlorhexidine. The total of this measures was effective in reduction the rates of bloodstream infectionassociated withcentral venous catheter (BI-ACVC).

## Conclusion

An educational program implementation to the control of the bloodstream infection associated with central venous catheter was effective in the intensive care unit of adults.

## Disclosure of interest

None declared.

